# Design and Validation of a Novel Method to Measure Cross-Sectional Area of Neck Muscles Included during Routine MR Brain Volume Imaging

**DOI:** 10.1371/journal.pone.0034444

**Published:** 2012-04-03

**Authors:** Alixe H. M. Kilgour, Deepak Subedi, Calum D. Gray, Ian J. Deary, Stephen M. Lawrie, Joanna M. Wardlaw, John M. Starr

**Affiliations:** 1 Geriatric Medicine Unit, Centre for Cognitive Ageing and Cognitive Epidemiology, University of Edinburgh, Edinburgh, United Kingdom; 2 Clinical Neurosciences, Brain Research Imaging Centre, University of Edinburgh, Edinburgh, United Kingdom; 3 Clinical Research Imaging Centre, Queen's Medical Research Institute, University of Edinburgh, Edinburgh, United Kingdom; 4 Scottish Imaging Network, A Platform for Scientific Excellence (SINAPSE), Edinburgh, United Kingdom; 5 Division of Psychiatry, University of Edinburgh, Edinburgh, United Kingdom; Institution of Automation, CAS, China

## Abstract

**Introduction:**

Low muscle mass secondary to disease and ageing is an important cause of excess mortality and morbidity. Many studies include a MR brain scan but no peripheral measure of muscle mass. We developed a technique to measure posterior neck muscle cross-sectional area (CSA) on volumetric MR brain scans enabling brain and muscle size to be measured simultaneously.

**Methods:**

We performed four studies to develop and test: feasibility, inter-rater reliability, repeatability and external validity. We used T1-weighted MR brain imaging from young and older subjects, obtained on different scanners, and collected mid-thigh MR data.

**Results:**

After developing the technique and demonstrating feasibility, we tested it for inter-rater reliability in 40 subjects. Intraclass correlation coefficients (ICC) between raters were 0.99 (95% confidence intervals (CI) 0.98–1.00) for the combined group (trapezius, splenius and semispinalis), 0.92 (CI 0.85–0.96) for obliquus and 0.92 (CI 0.85–0.96) for sternocleidomastoid. The first unrotated principal component explained 72.2% of total neck muscle CSA variance and correlated positively with both right (r = 0.52, p = .001) and left (r = 0.50, p = .002) grip strength. The 14 subjects in the repeatability study had had two MR brain scans on three different scanners. The ICC for between scanner variation for total neck muscle CSA was high at 0.94 (CI 0.86–0.98). The ICCs for within scanner variations were also high, with values of 0.95 (CI 0.86–0.98), 0.97 (CI 0.92–0.99) and 0.96 (CI 0.86–0.99) for the three scanners. The external validity study found a correlation coefficient for total thigh CSA and total neck CSA of 0.88.

**Discussion:**

We present a feasible, valid and reliable method for measuring neck muscle CSA on T1-weighted MR brain scans. Larger studies are needed to validate and apply our technique with subjects differing in age, ethnicity and geographical location.

## Introduction

Low muscle mass secondary to disease and ageing is an important cause of excess mortality and morbidity [1]–[4]. Studies investigating correlates of muscle loss or potential interventions to slow or reverse muscle loss require accurate measurements of muscle size. Current imaging techniques used to measure muscle size include whole body or regional DEXA scans and volumetric or cross-sectional area measurements on MR or CT scans of the arm or leg [5]. Arm and thigh cross-sectional area (CSA) have been used in previous studies as they are large and are viewed to be used in everyday tasks. However thigh muscle CSA has been shown to correlate well with total muscle mass and it maybe that other muscle groups around the body are equally useful as a guide of general muscle bulk [6], [7]. Whilst the above techniques remain the current gold standard, they are not commonly employed in clinical practice or in studies out with those directly investigating muscle mass (eg studies of sarcopenia or cachexia). Volumetric MR brain scans are commonly used in both research and clinical practice. These scans often include much of the posterior neck muscles. A technique to measure posterior neck muscle CSA on volumetric MR brain scans would therefore enhance the value of volumetric MR brains scans: both brain and muscle size could be measured without additional scanning.

Recent studies have shown a correlation between grip strength and cognition, which has implications for studying rates of ageing [8], [9]. However, few studies have investigated the relationship between muscle size and brain size. This is likely due in part to the fact that two different scans would be required in each subject to obtain these data. Both brain and muscle size are known to decrease with age, therefore studying the pattern of their inter-relationship would allow investigation of their shared risk factors which, in turn, may suggest underlying mechanisms. Many longitudinal aging studies include a volumetric MR brain scan [10]–[12]. If it were possible to measure muscle CSA reliably from volumetric MR brain scans and this measure was representative of general body muscle bulk, the relationship between muscle and brain size could be investigated using a single scan.

MR measurement of neck muscle cross sectional area (CSA) has been shown to be feasible in young healthy adults using scans dedicated to this purpose (ie MR Imaging of the neck), but older adults have not been studied [13]–[15]. These studies have demonstrated good inter-rater reliability [14], [15]. Moreover, we found no previous studies documenting a technique to measure neck muscle size on MR brain scans. The limited data that are available suggest that neck muscle CSA and strength are correlated, indicating that neck muscle CSA has good construct validity [16]. We aimed to establish a novel method for measuring neck muscle CSAs from routine MR brain volume acquisitions. This paper details the technique we developed and further studies to test its reliability, validity and repeatability.

## Methods

### Study 1: Feasibility study

#### Goal

To investigate whether it is feasible to measure neck muscle CSA on MRI volumetric brain scans.

#### Ethics & Sample

The volumetric MR brain scans used in this study had already been performed as part of the Lothian Birth Cohort 1936 (LBC) study, as a primary outcome for that study was brain volume measurement. Ethics permission for the LBC1936 study protocol was obtained from the Multi-Centre Research Ethics Committee for Scotland (MREC/01/0/56) and from the Lothian Research Ethics Committee (LREC/2003/2/29) and covers this sub-study because the ethics approval included the use of the data for future research purposes. The research was carried out in compliance with the Helsinki Declaration. All participants gave written, informed consent. Twenty consecutive scans from a final total of 735 were selected between 02/02/09 and 30/03/09. Participants were community-resident, all born in 1936 and without any known major musculoskeletal disease. Height, weight and grip strength in both hands were measured by trained research nurses at a clinical research facility [11].

#### Imaging Protocol

The MR imaging was performed with participants in the supine position on a 1.5 tesla MR imaging unit (Signa HDxt, GE Healthcare, Milwaukee, USA) at the Brain Research Imaging Centre (www.bric.ed.ac.uk). A phased array eight channel head coil was used and inversion recovery prepared volumetric T1 weighted images were acquired on a coronal plane for each patient. For this set of images, the alignment was perpendicular to the long axis of the hippocampus determined from a preliminary T2 weighted sagittal sequence. The flip angle was 8°, bandwidth 15.63 KHz, echo time (TE) 4 ms minimum to 13 ms maximum, repetition time (TR) 9.6 ms and inversion or preparation time (TI) 500 ms. The field of view (FOV), fixed superiorly at the cranial vertex, was 25.6 cm×25.6 cm, slice thickness 1.3 mm with no slice gap leading to 160 slices, displayed on a 192×192 matrix. These images took 8.13 minutes to acquire per patient. Full details of imaging protocol [17].

#### Development of neck muscle cross sectional area measurement technique

The image data were transferred to a Kodak Carestream picture archiving and communication systems (PACS) workstation where 3-D multiplanar reconstructions were performed. Freehand cursor was used to draw a region of interest (ROI) around the neck muscle of interest in the axial plane to obtain the cross sectional area on each side separately.

**Figure 1:**
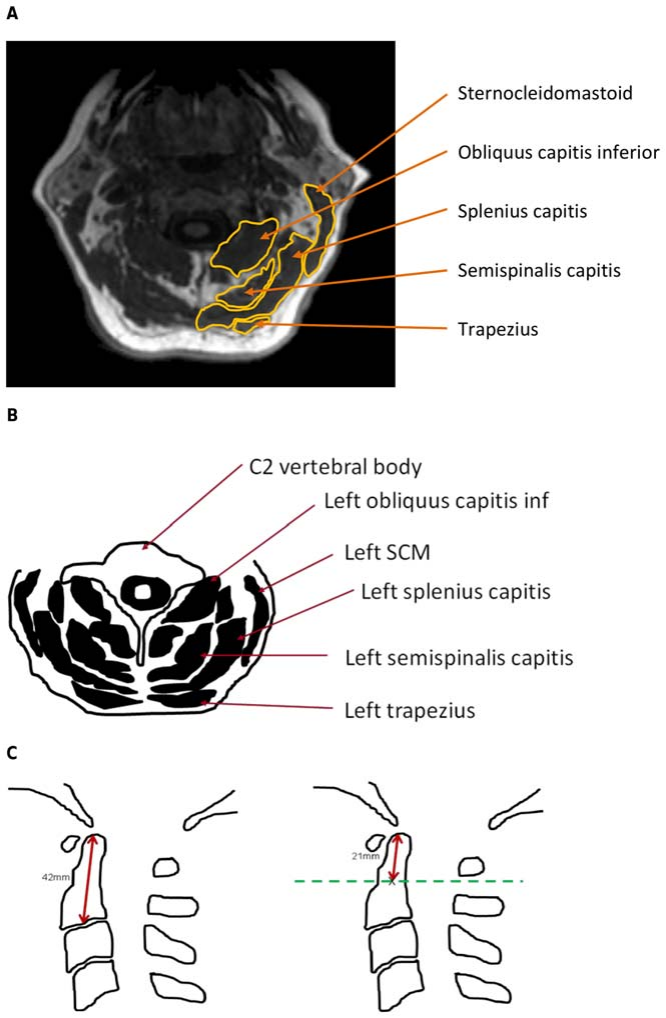
Figure of the posterior neck muscles and diagram demonstrating how the measurement plane was selected. A. Non-contrast T1-weighted MR of transverse plane of the neck at mid-infero-superior-C2 level. B. Outline diagram showing the neck muscles whose cross-sectional areas were measured. C. Outline diagram demonstrating how measurement plane is selected with an example C2 height of 42 mm.

Two raters tested feasibility in ten of the participants. We sought to develop a technique that ensured raters found the same level from which to make their CSA measurements. Our first attempt involved finding the MR slice in which the CSA of the obliquus capitis inferior was at its maximum in the axial plane. We chose the obliquus capitis inferior because it is a short muscle and its width varies more along its length than the other neck muscles in the scan. We then measured the CSAs for the largest muscles in that slice of the scan; sternocleidomastoid (SCM), obliquus capitis inferior, semispinalis capitis, splenius capitis and trapezius for both right and left sides (Figure 1). Although it was possible to measure the CSA of neck muscles using this technique, there were occasional large discrepancies between raters indicating that this method lacked reliability, particularly with regard to finding the level to take the measurements.

Therefore, in the second attempt, we measured the neck muscles' CSAs of a further ten participants, but this time we started with the images in the sagittal view with the volume images loaded into the multiplanar reformat view. We chose the slice where the C2 vertebral body height was at its maximum. We then identified the midpoint of the C2 vertebral body height including the odontoid by measuring along its vertical length from the odontoid tip to lower end plate using the cursor and then marked the midpoint. We then switched to the axial view of the multiplanar reformat at the vertical midpoint of C2 and measured the CSAs of the neck muscles in that axial image.

**Figure 2:**
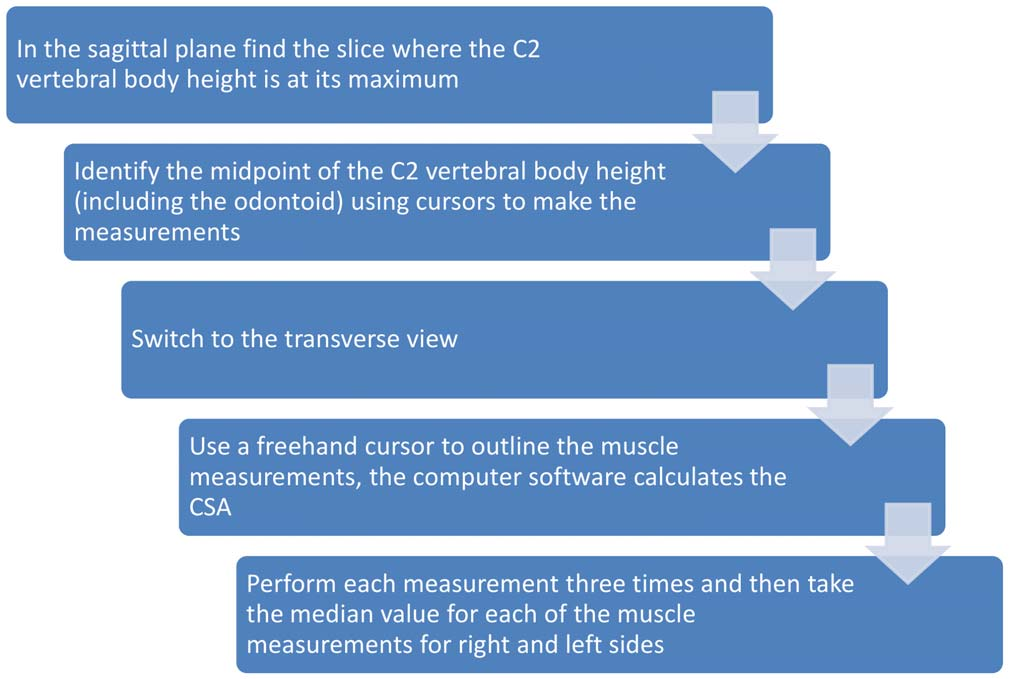
Flowchart summarizing method to measure craniad muscles cross-sectional areas.

We initially attempted to standardise the plane of the axial image on a line parallel to C2 end plates, but the variability of tilt in endplates meant that occasionally that line could go as high as suboccipital level posteriorly and thereby miss the muscles of interest. Setting the axial slice perpendicular to the vertical line of measurement through C2 did not work either as it proved difficult to manipulate the axial slice by small angle changes precisely enough. Therefore, we used the midpoint of C2 in the sagittal plane while viewing the images in the multiplanar reformat and then clicked on the corresponding axial image. This resulted in the axial slice being parallel to the lower border of the volume scan, but not related to any particular line in the participant. This time we measured the three posterior neck muscles (trapezius, splenius capitis and semispinalis capitis) individually and in combination. See Figure 2 for the chosen method.

### Study 2: Study to measure inter-rater reliability

#### Goal

To investigate and quantify whether two raters using this technique would produce the same measurements.

#### Sample

A further 20 scans from the LBC 1936 study were studied in addition to the 20 from the feasibility study, to give us a total of 40 scans to measure with the newly-developed technique.

#### Neck muscle cross sectional area measurements

We performed the measurements with the chosen technique, as described in the feasibility study, on all 40 MRI scans.(Figure 2) Each scan was measured three times by both raters and the median value for C2 body height and each of the three muscle measurements (SCM, obliquus capitis inferior and combined trapezius, splenius capitis and semispinalis capitis) were recorded separately for right and left sides for analysis. We noted that, unlike thigh muscles, there was only minimal intramuscular fat in the images of these neck muscles, so we did not seek to adjust for this or the small area of interfascial fat between trapezius, splenius capitis and semispinalis capitis in the combined group measure.

#### Analysis

To compare inter-rater reliability, we calculated the percentage difference in CSA as measured by the two raters and the two-way random effects absolute agreement intraclass correlation coefficients. Multiple linear regression analysis was used to estimate effects of sex and body mass index (BMI) on neck muscle CSA. Principal components analysis was used to extract a general trait for neck muscle CSA from the three individual muscle CSA measures: we accepted components with eigenvalues greater than unity and inspected the scree plot to identify the number of components. All analyses were performed using the SPSS 16.0 statistical package.

### Study 3: Study to measure repeatability of technique

#### Goal

To assess whether the technique would provide the same results on scans measured:

- on the same subject and the same MRI scanner on different days
- on the same subject and different MRI scanners on different days

#### Ethics

All subjects provided written consent and ethics approval was gained by the local ethics research committee for the original CaliBrain study (REC 05/S0801/105) [18], [19]. This included further use of the data for research purposes and therefore further ethics permission for this study was not required.

#### Sample

The CaliBrain study investigated the reliability of repeat volumetric brain MR measures with the same scanner and between different scanners [8]. We therefore used these data to test the reliability of repeat neck muscle CSA measurement from volumetric brain MR scans. The participants of the CaliBrain study were 14 normal volunteers from the three participating centres aged between 25 and 51 years, see below for details of the centres. As the data had been collected as part of the CaliBrain study no power calculations were carried out and we analysed all the available data. Exclusion criteria for the CaliBrain study were: previous history of a diagnosed neurological disorder or a major psychiatric disorder, treatment with psychotropic medication, including treatment for substance misuse and not meeting the MR safety criteria.

#### Imaging protocol

Each of the fourteen participants twice underwent a structural and functional MR brain scan at three imaging research centres around Scotland; The Department of Radiology, University of Aberdeen; The Brain Research Imaging Centre, Western General Hospital, University of Edinburgh; and The Neuroradiology Department, Southern General Hospital, NHS Greater Glasgow South University Hospitals Division. Therefore each participant underwent 6 separate scans. Each scan took place on a separate day and there were nominally 2 weeks between the scans at the same site. We only used the structural data for our study. The three scanners used were all manufactured by General Electric (GE Healthcare, Milwaukee, Wisconsin) and had primary field strengths of 1.5 T however the machines had differing software and hardware. Images were taken in the coronal plane at a slice thickness of 1.7 mm with no slice gap. 3D reconstruction was used to make the measurements with our technique. Further details of the imaging protocol can be found in the paper by Moorhead et al. [19]

#### Cross-sectional area measurement technique

The measurements were performed using Analyze, the biomedical image analysis software (Mayo Foundation, Rochester, Minnesota, USA).

The CaliBrain images were aligned with the ACPC line, an anatomical line which runs between the superior surface of the anterior commissure and the center of the posterior commissure. The feasibility and reliability studies had used images which were perpendicular to the MR table. Therefore the images underwent pre-processing prior to the measurements being made. This involved the images being tilted 15 degrees forward on the axial axis. This was actually preferable to the original study where the angle of the brain as viewed on PACS was not standardised, but just depended on how the patient placed their head in the scanner, as all the images in the CaliBrain study were standardised to an anatomical landmark, the ACPC line. Neck muscle CSA was measured as described in the feasibility study.

#### Analysis

ICCs were calculated for comparing within scanner and between scanner variations. When calculating the ICC for within scanner variation the measurements taken from the first and second scans were compared for each of the three individual measurements (ie combined group, SCM and obliquus) and the total neck muscle CSA (ie all three measurements for right and left sides added together). The ICC for between scanner variation were calculated using the mean total muscle CSA for each measurement on that site (eg (Total neck muscle CSA Edinburgh scan 1+Total neck muscle CSA Edinburgh scan 2)/2).

All data were analysed using the SPSS 17.0 statistics package. Three nonsynchronous sets of measurements were taken for each scan and the median values were used for the analysis.

### Study 4: External validity study

#### Goal

To assess and quantify whether neck CSA is related to mid-thigh CSA, which has been previously shown to be related to general muscle mass [6], [20].

#### Ethics

Ethics permission for the MR brain scans undertaken as part of the Lothian Birth Cohort (LBC) 1936 project had already been obtained as per study 1. A substantial amendment was submitted to allow us to recruit 25 subjects from the LBC 1936 pool and perform a MR scan of their mid-thigh. This was approved in April 2010.

#### Sample

735 LBC1936 participants had brain MR data available. Power calculations indicated that for a minimum Pearson correlation coefficient of 0.6 at alpha = 0.05, n = 20 provided 80% power and n = 26 provided 90% power. We therefore chose to scan 25 subjects. We contacted the subjects who had most recently had their MR brain scan, within a few weeks, to ensure that the effect of any variable on the thigh muscle bulk in the time lapsing between the MR brain and thigh scans was as small as possible. Participants were excluded if they had severe osteoarthritis affecting the knee or hip or a previous stroke, previous total hip replacement, or a history of any degenerative neurological disorder

#### Imaging protocol

The MR brain scans had been collected as part of the LBC 1936 study. See study 1 (above) for details of the protocol.

We used anatomical landmarks to identify the midpoint of the femur. We palpated for the protuberance of the greater trochanter and the upper border of the patella and then measured down the lateral aspect of the thigh using these landmarks and marked the midpoint. A cod liver oil capsule was then taped there to allow us to identify the corresponding MR slice. This was performed separately for each leg.

The scan was performed using a 3.0 tesla Siemens Verio research MR scanner (Siemens Medical, Germany) at the Clinical Research Imaging Centre within the Queen's Medical Research Institute. Images were acquired using a combination of body and spine matrix coil elements. The subjects lay supine for the scan. A coronal scouting scan was performed and then 5–10 axial images were taken with the cod liver oil capsules in the middle slices. Slice thickness was 3 mm with no slice gap.

#### Cross-sectional Area Measurements

Measurements for sternocleidomastoid, obliquus and the combined group (trapezius, splenius and semispinalis) were made using the above described technique on a PACS workstation.

The thigh muscle CSA measurements were also performed on a PACS workstation using the slice on each side where the cod liver oil capsule was at its widest which should indicate the anatomically chosen midpoint. Three measurements were taken on each leg: the anterior group, the medial group and the posterior group. The anterior group consisted of the quadriceps (vastus lateralis, intermedius and medialis and rectus femoris) and sartorius, the medial group of gracilis and the adductors (longus, brevis and magnus) and the posterior group of the hamstrings (biceps femoris, semitendinosus and semimembranosus).

Both the thigh and neck measurements were repeated 3 times for the left and right sides.

#### Analysis

All data were analysed using the SPSS 17.0 statistics package. Three nonsynchronous sets of measurements were taken for each subject and the median values were used for the analysis.

**Figure 3:**
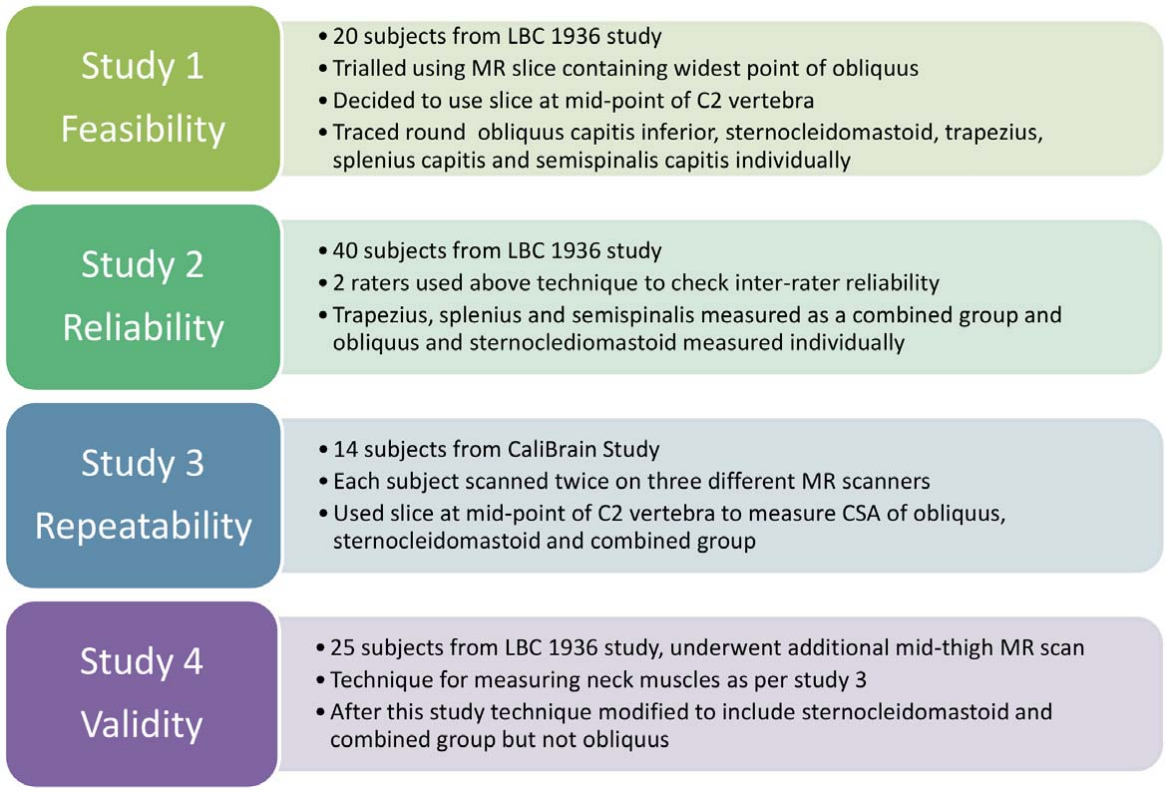
Diagram summarizing the methods for the four studies.

Please see figure 3 for a summary of the methods for the above four studies.

## Results

### Study 1: Feasibility study

The measurements made with the chosen technique were used to calculate intra-class correlation coefficients (ICC) to compare the median value of 3 measurements made by rater A against rater B. In this study we measured each of the three posterior muscles (trapezius, splenius and semispinalis) separately and together in a single measurement as a combined group. The boundaries between these three muscles are not clear and we thought that the differences in CSA measurements were a reflection of where the boundary was taken to be rather than true measurement differences in the size of the muscles themselves. The ICC and associated 95% confidence intervals support this view, as the values for the ICC for the three respective individual muscles were 0.78 (CI 0.16–0.94), 0.86 (CI 0.48–0.97) and 0.90 (CI 0.60–0.97) and the combined group ICC was much stronger at 0.99 (CI 0.95–1.00). Therefore we decided to use the combined measurement from thereon with individual measurements of the stand alone muscle, obliquus and sternocleidomastoid. For full results please see table 1.

**Table 1:** Intra-class correlation coefficients for the second technique trialled in the feasibility study (Study 1).

Measurement	ICC	95% CI
**Trapezius**	0.778	0.160–0.944
**Splenius**	0.861	0.475–0.965
**Semispinalis**	0.895	0.603–0.974
**Summation of Trapezius, Splenius & Semispinalis**	0.978	0.916–0.994
**Single measurement of combined group**	0.986	0.946–0.996
**Obliquus**	0.900	0.623–0.975
**SCM**	0.894	0.598–0.973

### Study 2: Study to measure inter-rater reliability

Of the 40 scans from the LBC 1936 cohort, one proved to be a duplicate and was excluded. One rater considered one scan to be unmeasurable whilst the other considered two scans unmeasurable. Scans were thus measured for 37 (18 male, 19 female) participants of mean age 72.0 (standard deviation 0.38) years when weighed and mean age 72.2 (sd 0.25) years when scanned. Men had a mean height of 1.73 m (sd 0.07), mean weight of 85.0 kg (sd 11.2) and mean BMI of 28.2 kg/m^2^ (sd 3.2). Women had a mean height of 1.59 m (sd 0.04), mean weight of 71.0 kg (sd 14.0) and mean BMI of 27.9 kg/m^2^ (sd 5.2).

**Figure 4:**
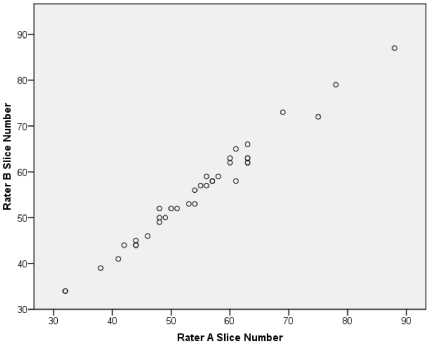
Plot of MR slice chosen as representing the mid-point of C2 for both raters.

Rater A measured mean C2 height as 3.7 cm (sd 0.5) in men and 3.6 cm (sd 0.2) in women. Rater B measured mean C2 height as 4.0 cm (sd 0.4) in men and 3.7 cm (sd 0.2) in women. These differences had no effect on slice chosen as midpoint of C2 for muscle CSA measurement (Figure 4).

Table 2 shows the mean CSAs per rater, absolute mean difference and mean difference as percentage of CSA. Intraclass correlation coefficients between raters were 0.99 (95% confidence intervals 0.98–1.00) for the combined group CSA, 0.92 (95% C.I. 0.85–0.96) for obliquus CSA and 0.92 (95% C.I. 0.85–0.96) for sternocleidomastoid CSA (Table 3). Obliquus CSA was predicted by sex (beta = −0.54 for women, p<.001) and BMI (beta = 0.36, p = .01) adjusted R^2^ for model = 0.40, sternocleidomastoid CSA by sex (beta = −0.60 for women, p<.001) and BMI (beta = 0.41, p = .001) adjusted r^2^ for model = 0.52, and combined CSA by sex (beta = −0.74 for women, p<.001 only) adjusted r^2^ for model = 0.55.

**Table 2:** Mean cross-sectional areas (CSAs) as measured by each rater summed for left and right, together with absolute mean difference and mean difference as percentage of CSA between raters (Study 2).

	Combined Group	Obliquus Capitis Inferior	Sternocleidomastoid
**Mean CSA rater A (mm^2^)**	1850	773	422
**Mean CSA rater B (mm^2^)**	1847	753	376
**Mean inter-rater difference (95% CI) (mm^2^)**	3 (−30, 36)	20 (−33, 73)	46 (−29, 63)
**Mean difference as percentage of mean CSA (95% CI)**	0.3 (−1.5, 2.0)	4.1 (−6.3, 14.4)	11.3 (7.1, 15.5)

**Table 3:** Intra-class correlation coefficients with 95% confidence intervals for the reliability study (Study 2).

Muscle Measurement	ICC	95% Confidence Intervals
**Combined group**	0.99	0.98–0.995
**Obliquus capitis inferior**	0.92	0.85–0.96
**SCM**	0.92	0.85–0.96

There were no significant associations between inter-rater CSA difference and mean CSA for the combined group (r = 0.08, p = .66), but larger inter-rater differences were significantly associated with smaller CSAs for both obliquus (r = −0.61, p<.001) and sternocleidomastoid (r = −0.39, p = .018). CSAs all correlated highly significantly with each other (p<.001): combined-obliquus (r = 0.59), combined-sternocleidomastoid (r = 0.66), obliquus-sternocleidomastoid (r = 0.50).

**Figure 5:**
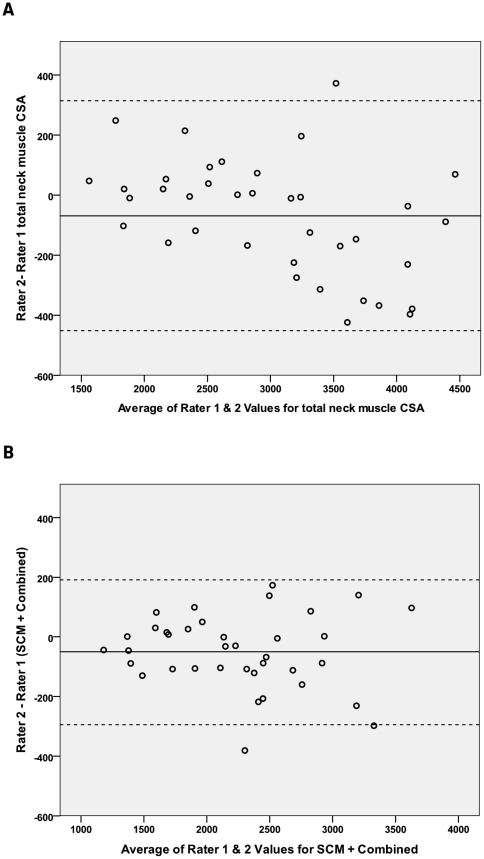
Bland-Altman plots for total neck muscle CSA and SCM+combined CSA measured by 2 raters. Bias of measurement between 2 different raters (mean of the ordinate) and limits of agreement (2sd) are represented by a solid and two dashed lines respectively. A. Bland-Altman plot for measurements of total neck muscle CSA by 2 different raters. B. Bland-Altman plot for measurements of SCM+combined CSA by 2 different raters.

A Bland-Altman plot for total neck muscle CSA demonstrates a degree of linear bias with Rater 2 reporting bigger measurements for the small neck muscle CSAs and smaller measurements for the bigger neck muscle CSAs (Figure 5A). If obliquus is removed, leaving the combined group plus SCM, this linear bias appears to resolve (Figure 5B). However the bias of measurement is small for both graphs.

Table 4 shows coefficients of variation (CV) for both raters and a Levene's test for homogeneity which found no significant difference between the CV for the two raters for any of the muscle measurements.

**Table 4:** Coefficients of variation (%) with 95% CI and Levene's significance test between the two raters (Study 2).

Measurement	Rater	Coefficients of Variation (CV) (%)	95% CI for CV	Levene's test (Significance)
**Combined group**	1	28.1	22.9–36.5	0.96
	2	28.5	23.2–37.0	
**Obliquus**	1	43.8	35.6–56.9	0.10
	2	32.3	26.3–42.0	
**SCM**	1	31.0	25.2–40.3	0.82
	2	29.8	24.2–38.7	
**Total neck muscle CSA**	1	28.2	22.9–36.6	0.47
	2	25.9	21.1–33.6	

The first unrotated principal component explained 72.2% of total CSA variance for the three muscles (loadings were 0.89 for the combined group, 0.81 for obliquus and 0.85 for sternocleidomastoid) and correlated positively with grip strength of both right (r = 0.52, p = .001) and left (r = 0.50, p = .002) hands. The second principal component had an eigenvalue of 0.51.

### Study 3: Study to measure repeatability of technique

Data were analysed for all 14 participants. Thirteen of the participants had undergone all 6 scans and one had undergone 5 of the scans having not completed their second scan in one location. There were 10 men and 4 women. The mean age was 36.3 years (range 25–51).

Mean values (sd) for the measurements across all six scanners for right and left sides added together were: SCM 4.97 cm^2^ (1.11), combined group 20.12 cm^2^ (5.74), obliquus 9.88 cm^2^ (3.23) and total neck muscle CSA 34.97 cm^2^ (8.67). ICCs were calculated for within scanner and between scanner variability (Tables 5 & 6). Within scanner ICCs for the Edinburgh scanner used for studies 1, 2 and 4 ranged from 0.83 for SCM to 0.96 for the combined group.

**Table 5:** Between scanner intra-class correlation coefficients for the repeatability study (Study 3).

Groups	ICC	95% Confidence Intervals	Sig
**E, A & G Total means**	0.94	0.86–0.98	p<0.001
**E, A & G SCM means**	0.76	0.53–0.90	p<0.001
**E, A & G Comb means**	0.95	0.89–0.98	p<0.001
**E, A & G Obliq means**	0.78	0.56–0.92	p<0.001

**Table 6:** Within scanner intra-class correlation coefficients for the repeatability study (Study 3).

Groups	ICC	95% Confidence Intervals	Sig
**E1 & E2 Total**	0.95	0.86–0.98	p<0.001
**A1 & A2 Total**	0.97	0.92–0.99	p<0.001
**G1 & G2 Total**	0.96	0.86–0.99	p<0.001
**E1 & E2 SCM**	0.83	0.55–0.94	p<0.001
**A1 & A2 SCM**	0.80	0.48–0.93	p<0.001
**G1 & G2 SCM**	0.90	0.70–0.97	p<0.001
**E1 & E2 Comb**	0.96	0.88–0.99	p<0.001
**A1 & A2 Comb**	0.97	0.92–0.99	p<0.001
**G1 & G2 Comb**	0.96	0.88–0.99	p<0.001
**E1 & E2 Obliq**	0.93	0.79–0.98	p<0.001
**A1 & A2 Obliq**	0.83	0.56–0.94	p<0.001
**G1 & G2 Obliq**	0.83	0.53–0.95	p<0.001

Key for Table 5& 6:

• E = scan performed in Edinburgh.

• A = Scan performed in Aberdeen.

• G = Scan performed in Glasgow.

• 1 & 2 = 1^st^ and 2^nd^ scan on that site.

• SCM = Sternocleidomastoid.

• Comb = Combined group (Trapezius, Splenius capitis, Semispinalis capitis).

• Obliq = Obliquus Capitis Inferior.

• Total = Total neck muscle CSA.

**Figure 6:**
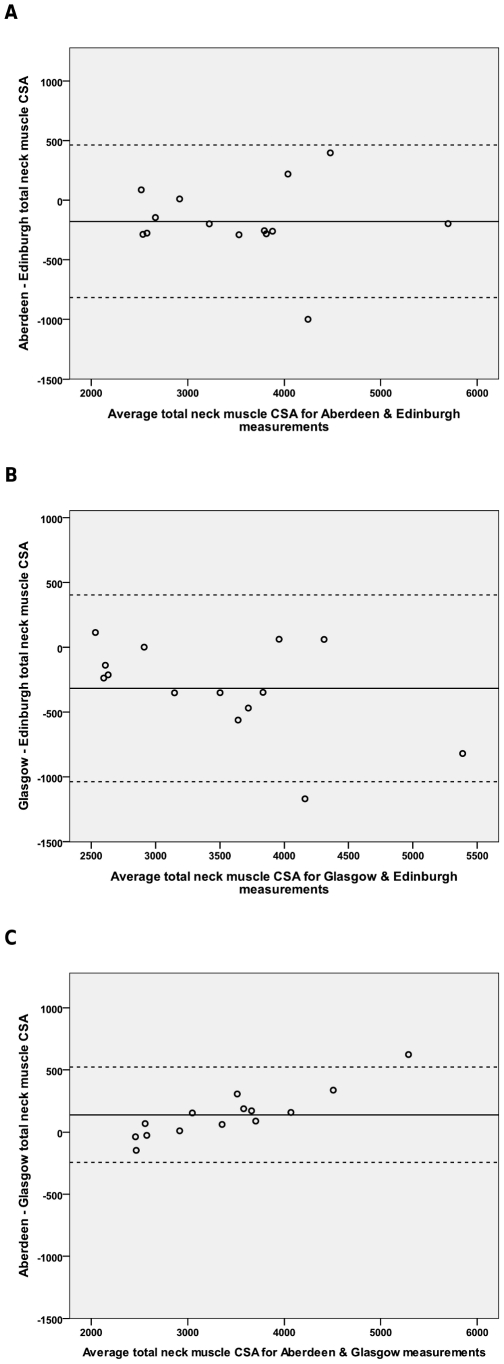
Bland-Altman plots for total neck muscle CSA measured on 3 different MRI scanners. Bias of measurement between different MRI scanners (mean of the ordinate) and limits of agreement (2sd) are represented by a solid and two dashed lines respectively. A. Bland-Altman plot for total neck muscle CSA measured on the Aberdeen and Edinburgh MR images. B. Bland-Altman plot for total neck muscle CSA measured on the Edinburgh and Glasgow MR images. C. Bland-Altman plot for total neck muscle CSA measured on the Glasgow and Aberdeen MR images.

Bland-Altman plots show no definite linear bias between the Edinburgh and Glasgow scanners and the Aberdeen and Edinburgh scanners (Figures 6A & 6B). The Aberdeen-Glasgow plot indicates that the Aberdeen scanner may overestimate larger neck muscle CSA and underestimate smaller neck muscle CSA (Figure 6C). However the numbers involved in this study were small (n = 14).

Table 7 shows the coefficients of variance (CV) for the mean values for each of the three scanners and a Levene's test of homogeneity which found no significant difference in CV for any of the three scanners, for any of the muscle measurements.

**Table 7:** Coefficients of variation (%) with 95% CI and Levene's significance test between the three MRI scanners (Study 3).

Measurement	Scanner	Coefficients of Variation (CV) (%)	95%CI for CV	Levene's test (Significance)
**Combined**	Aberdeen	32.2	23.3–51.9	0.89
	Edinburgh	27.1	19.7–43.7	
	Glasgow	26.0	18.9–41.9	
**Obliquus**	Aberdeen	30.6	22.2–49.3	0.53
	Edinburgh	36.7	26.6–59.1	
	Glasgow	28.1	20.4–45.3	
**SCM**	Aberdeen	21.7	15.7–35.0	0.95
	Edinburgh	20.6	14.9–33.2	
	Glasgow	22.1	16.0–35.6	
**Total neck muscle CSA**	Aberdeen	26.4	19.1–42.5	0.84
	Edinburgh	25.5	18.5–41.1	
	Glasgow	22.3	16.2–35.9	

### Study 4: External validity study

25 subjects underwent the additional thigh scan; however, only 24 could be used in the analyses as one patient had not tolerated the full MR brain scan so we were unable to make the neck muscle CSA measurements. There was no overlap between subjects in study 2 and study 4. Of these 24 subjects, 11 were female and 13 male. Mean age (sd) was 73.8 years (0.27). Mean weight (sd) for the women was 63.2 kg (15.4) and for the men was 85.6 kg (10.9).

Mean total neck muscle CSA (sd) was 22.5 cm^2^ (3.7) for the female subgroup and 38.1 cm^2^ (6.3) for the male subgroup. Mean total thigh muscle CSA was 184.3 cm^2^ (36.5) for the female subgroup and 277.0 cm^2^ (31.3) for the male subgroup. An independent t test showed that both total neck muscle CSA (p<0.0005) and total thigh muscle CSA (p<0.0005) were significantly different between the female and male subgroups. The correlation coefficient for all subjects for total thigh CSA and total neck CSA was 0.88 indicating that each explained at least 77.4% of the variance of the other.

## Discussion

### Summary of findings

This study sought to develop a technique to measure neck muscle cross-sectional area on volumetric MR brain scans. An initial feasibility study led to the formation of the technique.(Figure 2) The reliability study then demonstrated that the technique had high inter-rater reliability for measurement of the CSA of the combined trapezius, splenius and semispinalis group in older adults. Obliquus capitis inferior and sternocleidomastoid CSAs are smaller muscles and measurements were less reliable between raters, though intraclass correlation coefficients remained high.

Study 3 demonstrated that the technique has good within scanner and between scanner repeatability. The confidence intervals for the measurements of the combined group and the total neck muscle area are quite narrow however the confidence intervals for the SCM and obliquus capitis inferior measurements are wider. This is because the cross-sectional areas of the SCM and obliquus muscles are smaller than either the combined or the total measurements. This means that any measurement errors will account for a greater proportion of the CSA than for muscles with a large area.

The obliquus is a short muscle whose cross-sectional area varies greatly over its length, unlike the other four muscles, as it has a wide belly and comparatively narrow tails. Our technique meant that in some subjects we were measuring across the belly of the obliquus and in some just the tail end. It also runs at an oblique angle and we identified that this angle varies between subjects. This meant that the area of obliquus we were measuring was an inexact proxy. For these reasons we conclude that the obliquus measurement should not be included in our technique. The ICCs are not as strong for the SCM as the combined group in the repeatability and reliability studies, which is likely a reflection of its small size. However as there are not the same intrinsic anatomical problems in measuring this muscle as there are with the obliquus and as total neck muscle CSA including the SCM appears to have stronger ICCs than the combined group alone, it is probably beneficial to include the SCM in addition to the combined group to provide a better estimate of general muscle bulk.

The final study was designed to measure the external validity of the technique. It shows that there is a strong correlation between neck muscle cross sectional area and thigh muscle cross sectional area, which is often used as a proxy for general muscle mass [21]–[23]. The percentage variance (ie r-squared) is 76.7% and the 1^st^ unrotated principal component of neck muscle CSA was found in the reliability study to explain 72.2% of variance. Extracting principal components is useful to reduce random measurement errors that might be associated with individuals' neck positions for example. The principal component correlated positively with grip strength, providing further support for neck muscle CSAs' validity as an index of sarcopenia. This means that posterior neck muscles can be used equally as well as thigh muscles as an index of general muscle bulk [6], [7].

### Previous research on quantifying muscle mass

Previous studies quantifying neck muscle CSA have only focused on young subjects and have used scans performed specifically for that purpose. They have however shown good reliability in the techniques used [14], [15]. We found no previous studies which measured neck muscle CSA on MRI scans on elderly subjects and none which used MRI brain scans for this purpose.

When considering the validity of using a cross-sectional measurement of muscle size to infer general muscle bulk we referred to previous studies on body composition. Studies investigating how differing muscle groups relate to each other have tended to compare upper and lower limb muscle mass alone [24]–[26]. We found no studies which compared muscle CSA, mass or volume between any other two or more areas of the body, including the neck. Three large studies on body composition suggest that the distribution of muscle between the upper and lower limbs varies with gender, age, height, weight and ethnicity [24]–[26].

### Strengths and limitations of the studies

Although there is no reason to suppose that this methodology is not applicable in younger adults and older adults (ie 75 y+), three of the studies were restricted to a narrow age cohort around 72 years old and the study of younger subjects only had a n = 14. The study participants were all community-resident volunteers and thus relatively healthy and were not diverse in terms of geography or ethnicity. The narrow geographical location of the subjects is important as it has been shown that anthropometric measurements vary across the UK. Bannerman et al collected data from residents of Edinburgh and compared their results with anthropometric reference data from South Wales and Nottingham. They found significant differences between the three groups confirming their hypothesis that anthropometric measurements vary across geographical area [27].

Skeletal muscle can be split into two groups; postural and phasic. Postural muscles have a larger percentage of type 1 fibres and show less fatigability. Phasic muscles are primarily involved in movement and have a higher proportion of type 2 muscle fibres. A feature of ageing muscle is that type 2 fibre width decreases more than type 1 fibre width, therefore the relation between neck CSA (mainly postural, ie more type 1 fibres) and thigh CSA (a mixture of postural and phasic) will change with age [28]–[31].

Despite not standardising the angle of the axial measurement slice relative to the patient, we still achieved very high inter-rater reliability. However, it is possible that the measurement variability would be larger in a longitudinal study if the patients were in different positions in the scanner on each occasion. Such differences are usually only slight because head, neck and back are passively supported during scanning leaving the neck muscles in a relaxed state. Lateral changes are unlikely to have a major effect because muscle CSAs are summed for both left and right so that reductions on one side could be compensated by the accompanying increase on the other. Such compensation does not apply to antero-posterior positioning; however, small differences in this plane are also unlikely to be important. A difference in angle of C2 between two repeated scans in the sagittal plane of 5° would result in a CSA difference of 0.4%, 10° increases CSA by 1.5% and 15° by 3.5% all within the limits for inter-rater SCM CSA difference; even a 20° angle increases CSA by only 6.4%, probably at the limit of utility of the technique to detect medium effect sizes. If reliability in longitudinal studies was not acceptable (i.e. mean differences in plane angles measured at C2>20°), increased positional standardization would be necessary which might include using more than one anatomical marker from a T1-weighted volume scan for standardization.

Most of these limitations could be addressed by a larger study which included a wider spread of age, geographical area, ethnicity, health status and an equal gender balance. It would be interesting to look at muscles from elsewhere in the body also. For example including a measure of upper arm CSA and calf muscle CSA and to investigate how the comparative size of these muscles varies with age.

### Implications for future research

This new technique is particularly interesting because several of the longitudinal studies investigating ageing involve an MR brain scan, therefore the method could be used to measure changes in muscle size and consequently estimate sarcopenia in these studies without any further imaging. This will allow the wealth of variables already collected as part of these studies to be researched as possible correlates of sarcopenia. Longitudinal studies are important sources of information for researchers interested in age associated disease to allow identification of key risk factors. These in turn allow hypotheses to be generated which can lead to both an understanding of the mechanisms underlying these diseases, which may lead on to development of treatments, and the possibility of generating advice to prevent or slow down some of the disease processes. Although we developed the technique on MR volume brain images, it is now common to acquire volume data when performing a CT brain scan and, as there is often good differentiation between muscle and fat in the neck, the same approach could possibly work on CT scans as well. Further testing is required.

We now plan to use this technique on two longitudinal studies to allow us to investigate correlates of sarcopenia and identify possible causative factors from lifestyle and biomedical data which have been collected concurrently with the MRI scans.

### Conclusion

We have developed a feasible, valid and repeatable method for measuring neck muscle cross-sectional area on MR brain scans which has good inter-rater reliability. This technique can be used to measure neck muscle CSA which can serve as a proxy measure of muscle bulk as shown by the above factor analysis and shared variance measures. We have demonstrated that neck muscle CSA correlates strongly with grip strength, a commonly used functional measure. The development of a reliable method to measure neck muscle CSA from volumetric MR brain scans potentially opens up a new field of radiological aging research. This in turn will allow sarcopenia to be investigated in studies which have involved a brain scan but no measure of muscle bulk without involving any additional scanning. Additional studies are needed to investigate these important relationships further with particular reference to how the relationships change with age.
